# Supplementary material for: Diagnosis of urinary tract infections in the hospitalized older adult population in Alberta
Source: PLoS One. 2024 Jun 7;19(6):e0300564. doi: 10.1371/journal.pone.0300564 (PMC11161040; doi:10.1371/journal.pone.0300564)
Supplement: S1 Table — (DOCX) [file pone.0300564.s002.docx]

|  | *# of prescribed medications* | *Sex* | *Pre-admission Living Situation* | *History of major neurocognitive disorder* | *History of recurrent UTIs* | *Admitting Specialty* | *Delirium* |
| --- | --- | --- | --- | --- | --- | --- | --- |
| *Age* | 0.055 | 0.224 | **<0.001** | **<0.001** | 0.374 | 0.069 | **0.007** |
| *# of prescribed medications* |  | 0.785 | **0.010** | 0.052 | 0.934 | 0.055 | **0.038** |
| *Sex* |  |  | **0.040** | **0.049** | 0.092 | 0.214 | 0.162 |
| *Pre-admission Living Situation* |  |  |  | **<0.001** | 0.998 | 0.472 | **0.011** |
| *History of major neurocognitive disorder* |  |  |  |  | 0.061 | 0.106 | **<0.001** |
| *History of recurrent UTIs* |  |  |  |  |  | 0.346 | 0.189 |
| *Admitting Specialty* |  |  |  |  |  |  | 0.358 |

Table S1. Correlation matrix: test of multicollinearity.
