# Supplementary material for: Diagnosis of urinary tract infections in the hospitalized older adult population in Alberta
Source: PLoS One. 2024 Jun 7;19(6):e0300564. doi: 10.1371/journal.pone.0300564 (PMC11161040; doi:10.1371/journal.pone.0300564)
Supplement: Attachment — Submitted filename: Response to Reviewer Comments Feb 2024.docx [file pone.0300564.s003.docx]

# **Re: Diagnosis of Urinary Tract Infections in the Hospitalized Older Adult Population in Alberta**

Dear Editors:

Thank you for reviewing and recommending revisions of our manuscript.

We were appreciative to receive the constructive feedback and the opportunity to improve our manuscript. We have done our due diligence to review and consider each point made by yourselves and the two reviewers, and provided clarification as well as revisions in a transparent way. We feel that the quality of our paper has significantly improved because of your comments and our revisions.

We have replied to your comments, and that of the reviewers, point by point. You will find each review point by “reviewer comment”, and our response by “authors’ reply”. We have submitted a clean copy of our revised manuscript, along with a copy of the revised manuscript with tracking.

Sincerely,

Dr. Frances Carr on behalf of the authors

**Response to Editors comments**

1. Please ensure that your manuscript meets PLOS ONE's style requirements, including those for file naming.

*Authors: The manuscript has been modified to meet PLOS ONEs style requirements.*

2. Note from Emily Chenette, Editor in Chief of PLOS ONE, and Iain Hrynaszkiewicz, Director of Open Research Solutions at PLOS: Did you know that depositing data in a repository is associated with up to a 25% citation advantage (<https://doi.org/10.1371/journal.pone.0230416>)? If you’ve not already done so, consider depositing your raw data in a repository to ensure your work is read, appreciated and cited by the largest possible audience. You’ll also earn an Accessible Data icon on your published paper if you deposit your data in any participating repository (<https://plos.org/open-science/open-data/#accessible-data>)

*Authors: Thank you for the information, however, we feel this is not required at this time.*

3. Please include a separate caption for each figure in your manuscript.

*Authors: Thank you, this has been done.*

4. We notice that your supplementary table are included in the manuscript file. Please remove them and upload them with the file type 'Supporting Information'. Please ensure that each Supporting Information file has a legend listed in the manuscript after the references list.

*Authors: Thank you, the supplementary table has been removed from the manuscript and uploaded as supporting information.*

5. Please include captions for your Supporting Information files at the end of your manuscript, and update any in-text citations to match accordingly. Please see our Supporting Information guidelines for more information: <http://journals.plos.org/plosone/s/supporting-information>.

*Authors: Thank you, this has been done.*

6. Please review your reference list to ensure that it is complete and correct. If you have cited papers that have been retracted, please include the rationale for doing so in the manuscript text, or remove these references and replace them with relevant current references. Any changes to the reference list should be mentioned in the rebuttal letter that accompanies your revised manuscript. If you need to cite a retracted article, indicate the article’s retracted status in the References list and also include a citation and full reference for the retraction notice.

*Authors: Thank you, this has been done.*

**Response to Reviewers’ Comments**

**Reviewer #1:**

**The title of this manuscript is very well chosen and highlights the value of scientific data. The language is correctly used from grammatical point of view. Statistical data are very well processed, having statistical value.**

*Authors: Thank you for your comment.*

**Reviewer #2:**

**General Impression: This publication offers insightful information about the misdiagnosis of urinary tract infections in hospitalized elderly persons. It has a clear introduction, objectives, methodology, results, and conclusions and is well-organized. The study's primary focus is succinctly summed up in the title. A thorough description of the study's design, data gathering procedures, and statistical analysis is given in the Methods section. The statistical analysis section is well-described, outlining the initial exploration, descriptive analysis, and logistic regression modeling. The Results section has a clear, informative organization. The important conclusions of the study are succinctly summarized in the Conclusion section, along with their obvious implications and potential directions for further research. It adds to our understanding of UTI diagnosis in older persons and is in line with the goals of the study. Overall, the manuscript presents a methodologically solid scientific study with evidence to back up its findings. consider these few minor issues:**

*Authors: Thank you for your feedback!*

**Minor Issues:
1. Background: Include recent references to emphasize the current relevance of the problem. Most of the references are too old.**
*Authors: Thank you for your feedback. Several of the references are quite old, and whilst we have replaced some of the oldest references, the remaining references are thought by the authors to be necessary.*

**2. Chart Review: Indicate whether there were any standards for resolving differences between the reviewer and senior investigators as well as the process used to do so. It would be helpful to provide details on inter-rater reliability or any steps taken to guarantee consistency between senior investigators and the lead reviewer (SL).**

*Thank you for this observation. A description of the process for resolving conflict has been added. We did not perform a formal IRR analysis, but there were no patient records for whom extensive discussion was required, as we had defined very binary “present or not” standards for the documentation****.*
3. Statistical analysis (Line 144): Consider providing more details on the specific statistical tests used for initial exploration and descriptive analysis, as this will help readers better understand the analytical approach.**
*Thank you for this suggestion. We have listed the statistical tests used, but feel that a detailed description of the reasoning behind them beyond the scope of this paper. The tests used are all standard.*

**4. Multicollinearity (Line 149): It is mentioned that multicollinearity tests were performed on baseline, admission, and clinical parameters linked to a confirmed diagnosis of UTI. Provide a brief explanation of how multicollinearity was assessed and handled in the analysis**

*Thank for this suggestion. The explanation of multicollinearity has been added within the changes in response to point 3 above.*
